# Unconscious death thoughts: Do they play a role in time trade-off and visual analogue scale scores for health?

**DOI:** 10.1007/s11136-025-04145-0

**Published:** 2026-02-03

**Authors:** Peep Stalmeier, Fanni Rencz, Bastiaan Rutjens, Bram Roudijk

**Affiliations:** 1 Radboud Institute for Health Sciences, Nijmegen, The Netherlands; 2 Department of Health Policy, Corvinus University, Budapest, Hungary; 3 University of Amsterdam, Amsterdam, The Netherlands; 4 EuroQol Research Foundation, Rotterdam, The Netherlands

**Keywords:** Time trade-off, Visual analogue scale, Death thoughts, DTA, Terror Management Theory, I30, J17

## Abstract

**Background:**

Several factors influence the Time Trade-off (TTO) and Visual Analogue Scale (VAS) health measures. In qualitative TTO studies, respondents often report thoughts of death which may induce mortality awareness. According to Terror Management Theory (TMT), mortality awareness gives rise to anxiety and increases unconscious death thought accessibility (DTA), potentially mitigated by secure attachment. Therefore a relation between DTA and attachment versus TTO and VAS is expected.

**Research questions:**

**(**1) Does DTA occur in TTO and VAS methods, (2) How are DTA and attachment related to TTO and VAS scores. We hypothesize: (1) DTA increases when respondents complete TTO or VAS tasks, and (2) TTO scores increase in those with a secure attachment.

**Methods:**

In an online experiment, 4572 members of the general public were randomised to one of four conditions: TTO, VAS, Mortality Salient (MS), and control. TTO and VAS scores were obtained for a hypothetical wheelchair health state, and for self-experienced health. MS and control conditions served as manipulation checks. DTA was measured with a validated word completion task. ANOVAs and Pearson’s correlations examined differences between conditions and associations among variables.

**Results:**

Mean (SD) DTA scores were: TTO = 2.86, (1.75); VAS = 2.94, (1.72); MS = 3.24, (1.89); control = 2.98, (1.70). The MS condition showed elevated DTA. Unexpectedly, DTA in the TTO condition tended to be *lower* than in the control condition, 95% CI [− .27, .03]. DTA was not associated with the TTO. DTA was negatively correlated with the VAS, both for the wheelchair and self-experienced states (both r = − 0.13, *p* < .01). Attachment showed negative correlations with the TTO and VAS self-experienced health scores (− 0.17 to − 0.13, *p* < 0.001), but was not associated with hypothetical wheelchair scores.

**Conclusion:**

DTA and attachment are novel factors influencing TTO and VAS scores. Our data suggest that VAS may reflect broader psychological health concepts than the TTO, despite that both methods quantify health. The rational nature of the TTO may suppress DTA. In this online context, DTA was unrelated to hypothetical wheelchair state scores, suggesting that DTA might have little impact on national health valuation studies.

**Supplementary Information:**

The online version contains supplementary material available at 10.1007/s11136-025-04145-0.

## Introduction

In health care decision making, the value of health is measured using preference-based or judgement-based methods. A widely used preference based method is the Time Trade-off (TTO) method [1–6]. In this method, respondents sacrifice hypothetical life years in full health to avoid living in a lesser health state [1, 7]. As an example, consider the state ‘being in a wheelchair’. A respondent willing to sacrifice or trade 3 years in full health, is indifferent between 7 years in full health and 10 years in a wheelchair. Under certain assumptions, the value of living in a wheelchair then equals 0.7 [6, 8]. For more severe health states, sacrificing more life years would be expected, corresponding with lower TTO scores. As for judgement tasks, a commonly used task is the visual analogue scale (VAS) in which respondents judge the value of health on a 100-point scale, ranging, for instance, from worst to best imaginable health [9]. TTO scores are influenced by e.g., age, gender, and religion [10–13]. This informs the interpretation of QALY and cost-effectiveness analyses. Our study considers additional factors.

The TTO method entails comparisons with being dead [1, 14–17]. Health states are typically presented as lasting 10 years followed by death, or are valued as better or worse than dead [9, 18]. In TTO think-aloud studies, thoughts about death are often mentioned [16, 19]. Sometimes intense death thoughts are reported, e.g., “at the moment, I wouldn’t want to say that I want to die immediately but that might be because I can’t believe or imagine it.”[16]. Thoughts about death, according to Terror Management Theory (TMT), lead to anxiety and heightened death thought accessibility (DTA). The unconscious defense of one’s cultural worldview, together with self-esteem, and attachment, buffer mortality awareness [20–22]. Helm et al*.* provides a concise overview of TMT [23]. While previous qualitative studies have reported death-related thoughts in TTO, no study has systematically measured unconscious DTA in this context.

A conceptual study proposed a link between TMT and TTO scores, arguing that TMT defense strengths affect the number of years traded off in the TTO [11]. In particular, the value ‘prolonging life’ was used as a linking pin between TTO and TMT for two reasons: (1) upholding this value has been shown to elevate TTO scores, and (2) in TMT ‘prolonging life’ is a conscious defense, that reduces mortality awareness [11].

Our main aim is to assess if DTA is elevated in respondents confronted with the TTO and VAS tasks. TTO and VAS scores were measured for the health states, ‘living in a wheelchair’ and for ‘self-experienced health.’ Based on the propositions of TMT and the death related nature of the TTO and VAS tasks, two hypotheses are tested. Hypothesis 1: DTA is expected to be higher in TTO and VAS tasks compared to control conditions. Hypothesis 2: Individuals with stronger attachment styles will report higher TTO scores. [11] Secondary aims were to study the role of conscious and unconscious defenses in relation to TTO and VAS.

## Methods

### Design

**Fig. 1:**
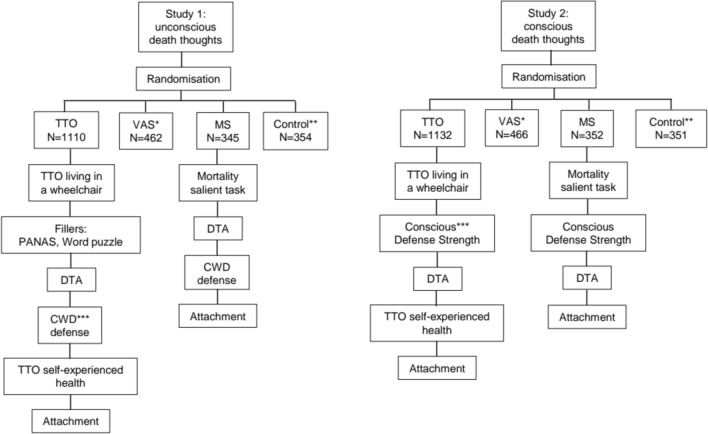
Experimental design: *VAS replaces the TTO, MS mortality salient task, **television control task replaces the mortality salient task, PANAS Positive and Negative Affect Schedule, DTA death thought accessibility, ***CWD Cultural Worldview Defense. For the measurement of CWD and conscious defense strengths, see "Appendix"

Two separate studies were designed to assess how TMT defenses were related to TTO and VAS scores. Study 1 included an unconscious defense measure, Study 2 included a conscious defense measure [17, 24]. Figure 1 presents a flowchart of the experimental design. Each study had four conditions: TTO, VAS, mortality salient (MS), and a control task, amounting to eight groups for the two studies combined. The MS task is used in TMT to induce death thoughts and serves here as a manipulation check to verify the sensitivity to detect DTA differences [17]. In a pilot in study 1, 300 respondents were randomized to MS and control groups and a reliable difference between MS and control conditions was detected. Remaining respondents were randomised in consecutive order to one of the eight groups without balancing. The studies received a waiver from the Ethical Commission East Netherlands, dossier nr. 2022-16000.

### Respondents

Respondents were part of a research panel of a commercial marketing bureau, Blauw Research, Rotterdam, the Netherlands. This being an explorative study, a representative sample of the Dutch adult general population was not sought. Data collection took place between 26th April 2023 and 3rd of June 2023. Respondents gave informed consent at the start of the survey and received a remuneration of 3.26 Euro’s. Missing responses were not accepted. The marketing bureau did not provide data on the number of respondents dropping out.

### Procedure

All questionnaires were in Dutch. The online self-administered survey started with a physical and mental health self-description using the EQ-5D-5L followed by the TTO in the TTO condition and the EQ-VAS instrument in the VAS condition [9, 25]. For respondents scoring high on anxiety/depression (> = level 4 in the EQ-5D-5L descriptive system), the survey was aborted because it was believed that the death related questions might trigger emotional distress. Next, the survey continued with socio-demographic questions on age, gender, working status, education, having kids, being religious, and believing in a life after death. For the TTO condition, the TTO for ‘living in a wheelchair’ was measured, followed by the measurement of DTA. Next followed measure of unconscious defense strength in Study 1 and a measure of conscious defense strength in Study 2. Next, the TTO for self-experienced health was measured. The survey ended with an attachment measure. For the VAS condition, the procedure was identical to the TTO condition with the TTO replaced by VAS. The sequence for the MS and control conditions were also identical with the MS replaced by the television control task. Durations of the survey and TTO tasks were recorded for quality control purposes.

### Four independent conditions

#### TTO

In this condition, respondents were confronted with the TTO. Practice questions were omitted on purpose to avoid learning and desensitization effects on DTA. TTO scores for ‘living in a wheelchair’ (TTOwheel) and self-experienced health (TTOown) were measured. The TTO was simplified and implemented as a downward titration task as full implementations of e.g., the composite TTO task showed poor results in online self-complete settings [26]. For the wheelchair TTO, respondents were asked to imagine they lived for 10 years with a mobility problem requiring the use of a wheelchair, followed by immediate death. The indifference procedure started with a comparison of living 10 years in a wheelchair (life A) and 10 years in full health (life B). Respondents were asked whether they preferred life A or life B using a forced choice. Subsequently, the number of years in full health was presented in descending order, in steps of one year, down to a comparison of life A with immediate death [27, 28]. For the measurement of TTOown, ‘living 10 years in a wheelchair’ was replaced by ‘living 10 years in your present health’. The wheelchair health state is widely used as a practice state in EQ-5D valuation studies. It is easy to imagine and typically not considered worse than dead.

#### VAS

In this condition, respondents were confronted with the EQ-VAS [9]. VAS values living in a wheelchair’ (VASwheel) and self-experienced health (VASown) were measured using the EQ VAS 100-point rating scale with ‘the best health you can imagine and ‘the worst health you can imagine’ as anchors [9, 29]. For the VASwheel, respondents were asked to imagine ‘living 10 years in a wheelchair’, and value this life on the rating scale. For self-experienced health, respondents were asked to rate their ‘own health today ‘ on this rating scale. 

#### MS task

The MS condition is used to induce death thoughts. It involves writing a short essay about one’s own death and uses two instructions: (1) describe the emotions you feel when you think about your own death, (2) imagine what will happen to you physically, when you die [15, 17].

#### Control task

Respondents are asked to write a short essay about their emotions and physical experiences while watching television. This task is frequently used in the TMT literature as a neutral condition [17].

#### Measurement of DTA

When death related stimuli are present, both conscious and unconscious death thoughts emerge. In order to measure the unconscious DTA component, the conscious death thoughts need to be dampened first [30]. For this purpose, delay or filler questions are used. After these preparations, unconscious thoughts are measurable.

#### Positive and negative affect schedule (PANAS)

The first filler questionnaire is the 20-item PANAS measure. It asks both about positive affects such as feeling enthusiastic or strong, and negative affects such as feeling afraid or hostile [31].

#### Word puzzle

The second filler questionnaire was a word puzzle task. Respondents had to find four words in a rectangle of nine by six letters. Respondents searched and reported these four words, and continued with the questionnaire.

#### Death thought accessibility (DTA) questionnaire

DTA is measured with the Dutch version of a validated word completion test and sensitive to TMT manipulations [32]. Sixteen Dutch ambiguous word fragments were chosen (in each fragment one letter was missing) [32]. Eleven of these fragments could be completed either as a death related or a neutral word (e.g., ‘doo.’, which could be completed as ‘dood’ (death), or ‘doof’ (deaf). The other five word fragments could only be completed in a neutral fashion e.g., ‘kas.’ can only be completed as ‘kast’ (cupboard). The DTA score therefore ranges from 0 to 11.

### Attachment

Attachment was measured using the validated Dutch version of the Experiences in Close Relationships [33]. This questionnaire comprises two scales: Attachment Anxiety and Attachment Avoidance. An example of an Attach Anxiety item is: ‘I’m afraid that I will lose the love of people around me.’ An example of a positively framed Attach Avoidance item is: ‘People around me really understand my needs.’ Higher Attachment Anxiety and Avoidance scale values indicate worse attachment. The questionnaire contains thirty-six items with a 7-point response scale ranging from ‘strongly disagree ‘ to ‘strongly agree’. For this study, the questionnaire was deemed too long and reduced to sixteen items, eight for each scale. Positively and negatively worded items were balanced as much as possible. Scale values were calculated summing the scores of the items within the attachment anxiety or attachment avoidance scales, reversing scores for positively framed items. In our study, Cronbach’s alpha for the Attachment Anxiety and Attachment Avoidance scales were 0.81, and 0.69, respectively.

### Conscious and unconscious defense strengths

The measurement of conscious and unconscious defense strengths is described in the "Appendix". Differences between MS and control conditions were anticipated based on the existing literature. If such differences were not found, these measures were discarded.

### Power calculation

Based on the Many Labs 4 replication study, *Chatard *et al*.* calculated an effect size of 0.27 of another TMT concept, that is cultural worldview defence (CWD) [34, 35]. This effect size was the basis for our power calculations, but now for the DTA measure. Because DTA has never been studied together with the TTO or VAS, we wanted to be able to detect effect sizes of 0.2, and correlations between TTO/VAS with DTA of 0.13, for study 1 and 2 each. With a significance level of 0.05, and a power of 0.8, this would necessitate about five hundred respondents for each condition per study.

For various sensitivity analyses involving the TTO, we anticipated that up to 50% of the TTO responses could not be used for the following reasons: 1) respondents may have more inconsistent TTO responses in an online survey, 2) analyses excluding respondents completing the survey in an unrealistic time frame. Therefore, the number of TTO respondents was raised to 1120. As a result, also considering the costs, for the TTO, VAS, MS, and control conditions, 1120, 466, 350, 350 respondents were envisaged, respectively, for study 1 and 2 each.

### Analysis

To answer the questions regarding DTA differences between the TTO, VAS, MS, and control conditions, ANOVAs were used with planned comparisons. Because the four conditions yielded six comparisons, significance levels were reduced by a factor of six (Bonferroni correction for multiple comparisons). Effect size was calculated using pooled variances.

TTO scores were calculated as y/10 where y was the number of years after which the preference switched from (y years in full health) to (10 years, wheelchair), or (10 years, self-experienced health). TTO scores were flagged as inconsistent if more than one preference switch occurred. To answer the questions regarding associations between DTA versus TTO and VAS scores, Pearson’s correlations were used, interpreted as weak, moderate, or strong for r = 0.1, 0.3, and 0.5, respectively [36]. For correlational analyses with the TTO, only values of consistent respondents were used. For reasons of quality control, separate analyses were done in a sample excluding non-traders, or excluding respondents with survey times below the 10th or above the 90th percentile. 

## Results

A total of 4572 respondents were included, for the TTO (N = 2242), VAS (N = 928), MS (N = 697), and control (N = 705) conditions. For surveys completed in less than 30 min, the average duration was 9.86 min (SD = 4.81 min, N = 4377). All respondents were used in subsequent analyses. General characteristics of the sample are presented in Table 1.

**Table 1:** General characteristics of the respondents

Characteristics	Overall (n = 4572) %
Age, years	49.0 (18.2) [Mean, (SD)]
18–34	28.1
35–44	15.2
45, and older	56.6
Sex, Female	50.3
Having kids	64.0
Highest finished education	
Low	21.2
Middle	33.7
High	45.2
Paid work	62.8
Religious	42.9
Belief in life after death	64.0

Our sample was older than the general population in the Netherlands, which has an average age of 42.4 years [37]. It contained more respondents having finished a middle educational level (for low, middle, and high educational level, the distribution in the Dutch population is 20, 44, 36.5%, respectively) [37]. In Studies 1 and 2, differences between the MS and control condition for the unconscious and conscious defense strengths were expected [38]. However, as these differences were not found, these two measures were discarded (see "Appendix").

Groups differed on education and belief in life after death. Corrected analyses are presented. Table 2 shows the mean DTA scores for the four conditions for the combined sample (Studies 1 and 2). The mean DTA scores differed across the four conditions (ANOVA F-test(3, 4568) = 8.62, *p* < .001). Pairwise comparisons, after Bonferroni correction of the p-values, showed that reliable differences occurred solely because the DTA in the MS condition was larger than any other condition: difference (MS—TTO) = 0.39, SE = 0.08, *p* < .001, 95% CI [0.24, 0.54]; difference (MS—VAS) = 0.30, SE = 0.09, *p* < .001, 95% CI [0.13, 0.47]; difference (MS—control) = 0.27, SE = .09, *p* = .004, 95% CI [.08, 0.45]. Thus, DTA in the TTO and VAS conditions was not elevated, contrary to our expectations based on reports of death thoughts in qualitative studies. The effect size of the difference between the MS and control difference was 0.15, thus passing the manipulation check. Sensitivity analyses for response times ranging between the 10th and 90th percentile, or omitting non-trading respondents, showed comparable results. When comparing study 1 and 2, the average DTA levels and differences between conditions were similar.

**Table 2:** Unconscious death thoughts (DTA) by condition

Condition	Mean (SD)	N
TTO	2.86 (1.75)	2242
VAS	2.94 (1.72)	928
MS	3.24 (1.89)	697
Control	2.98 (1.70)	705

TTO = Time trade-off, VAS = Visual Analogue Scale, MS = Mortality Salient, Control = television control. In the TTO condition, both consistent and inconsistent respondents are included. DTA is measured after the TTO or VAS task for the state ‘being in a wheelchair’

Of note, in Table 2, there is a trend towards a *lower* mean DTA in the TTO condition compared to the control condition, 2.86 and 2.98, respectively. This difference equals − 0.12 and the 95% CI of this difference equals [0.03, − 0.268]. This indicates that in future online experiments, the DTA in the TTO condition is not likely to exceed the DTA in the control condition by 0.03.

We now turn to the correlational analyses of the TTO and VAS scores versus the DTA and attachment measures in Table 3. For the TTOwheel scores, 1959 out of 2242 observations were consistent (87%) and could be used. For the TTOown score, 2023 out of 2242 observation were consistent (90%). In surveys completed in less than 30 min, the average duration of the TTOwheel task is 1.46 min (SD = 1.09 min, N = 2138), and 1.03 min (SD = 0.78 min, N = 2138) for the TTOown task.

In study 1, DTA is followed by a measure of cultural worldview defense (CWD). According to TMT, CWD reduces DTA to control levels [39]. Since TTOown and VASown are measured after CWD, this timing will compromise their associations with DTA. Therefore, only data from study 2, which does not have CWD defense, were used.

The VAS scores were significantly, though weakly, associated with DTA. In Study 2, attachment anxiety moderated the effect of DTA on the VASwheel score, *p* = .03. Further, TTOwheel and VASwheel scores were weakly related to Attachment Avoidance, however, these associations were not considered meaningful given the large sample size and their marginal significance level. Somewhat stronger associations between attachment with TTOown and VASown scores were found, all pointing in the negative direction. Results for sensitivity analyses were comparable.

## Discussion

This study introduces unconscious death thoughts into health economics, exploring whether (1) TTO or VAS tasks evoke unconscious death thought accessibility (DTA), and (2) how DTA and attachment relate to these tasks. The results show that DTA is not elevated in the TTO and VAS conditions. VAS scores were weakly and negatively associated with DTA. Self-experienced TTO and VAS scores were weakly and negatively associated with attachment anxiety and attachment avoidance.

As expected, the MS condition showed higher DTA than the control [17]. Our study thus passes an important TMT manipulation check. However, the DTA effect size between the MS and control condition was only 0.15, compared to typical effect sizes of 1.0 reported in the literature [32, 40, 41]. Unexpectedly, the DTA levels in the TTO groups were slightly lower than in the control groups. The 95% confidence interval indicated that the TTO condition is unlikely to exceed the control condition by 0.03. Given the standard deviation of the DTA scores, about 1.75, DTA in the TTO condition is unlikely to significantly exceed the DTA in the control condition, at least in this setting.

In retrospect, a psychological reason for lower DTA in the TTO condition may lie in dual-process theory, which makes a distinction between the conscious rational and the unconscious emotionally driven experiential system [42]. Properties of the rational system are e.g., analytic, logical, and encoding reality in abstract symbols, words, and numbers. Properties of the experiential system are e.g., holistic, affective, encoding reality in concrete images (for instance in dreams), metaphors, and narratives [42]. Because the TTO is a highly number oriented task, it is putatively processed by the conscious rational system. However, unconscious death thoughts occur in the experiential subconscious system. Hence, resources used up by the TTO task in the rational system might suppress DTA in the experiential system, as we found. Supporting this reasoning, prior research showed that inducing people to think about death rationally (vs. experientially) reduced the effect of MS on DTA [43]. Other explanations include desensitization due to repeated exposure to TTO questions; however, this was mitigated here by omitting TTO practice trials. Also, the TTO task may have led to cognitive overload. While cognitive overload was found to affect the time course of DTA emergence, it does not appear to influence its overall level. Furthermore, respondents may have engaged in emotional distancing, which is not engaging properly to avoid death thoughts. However, this seems to be unlikely given the verbal reports about death thoughts in qualitative TTO studies [39].

The literature about DTA triggers is mixed. The earlier TMT literature emphasized that stimuli had to be specific to induce CWD against DTA, which would argue against elevated DTA in the TTO condition.[17, 39]. For instance, thinking about intense physical pain, or the salience of concerns about the meaningfulness of life did not induce death thoughts.(*Baldwin and Wesley*, cited in [17]) In contrast, later literature reported a large variety of stimuli inducing CWD or DTA. For instance, three studies showed that considering the value or meaning of life induced DTA or CWD [41, 44, 45]. Four categories of stimuli inducing CWD are given in *Burke* or Hayes [21, 44]: (a) the death essay questions used in our study, (b) subliminal death primes, c) other questionnaires (e.g., a fear of death questionnaire), and (d) other (e.g., video or story with death themes) [21, 44]. These stimuli gave rise to more or less equally sized CWD [44]. Our, by now disproved, expectation of elevated DTA in the TTO condition rested on such reports. However, our findings show that the TTO is simply not such a stimulus despite the reports of death related thoughts in qualitative TTO studies.

As for associations, DTA was not related with the TTO, supporting our reasoning that the rational nature of the TTO suppresses DTA. For the VAS, higher DTA leads to a lower VAS score for the ‘living in wheelchair’ and ‘self-experienced health’ states. The latter seems intuitively clear from a quality of life point of view and is also corroborated in a clinical population showing that greater DTA lowers emotional well-being [46]. The negative association with the 'wheelchair' VAS scores is not intuitive and further investigation is needed.

Furthermore, associations with attachment anxiety and attachment avoidance were examined. These attachment styles both indicate worse attachment. These two styles were weakly and negatively associated with the TTO and VAS scores for self-experienced health, but not for hypothetical states. On the one hand, the negative associations of attachment styles with TTO and VAS scores for self-experienced health can be interpreted as reflecting a loss in quality of life caused by a worse attachment style [47, 48]. On the other hand, the negative association may be considered to support a recently proposed framework explaining TTO scores [11]. According to this framework, a decreased defense strength in TMT, here worse attachment, is hypothesized to be associated with lower TTO scores, which aligns with our findings [11, 49]. Thus, attachment is correlated with the TTO for the self-experienced state, but not the hypothetical state. A similar finding was reported previously for self-esteem in a clinical population [50]. A psychological explanation is that valuing self-experienced health is more emotionally charged, or may put respondents in a self-focused condition evoking thoughts such as ‘who am I’ or ‘what is important to me’ [51].

### Limitations

Following the TMT procedures, DTA was measured after the filler questionnaires for the TTO and VAS tasks for ‘living in a wheelchair.’ However, DTA was measured before the TTO and VAS for ‘self-experienced health.’ The latter could affect associations though similar correlations with DTA across VASwheel and VASown scores suggest minimal impact. (see Table 3). Another limitation ìs that conscious and unconscious DTA measures, see "Appendix", were not significant and were therefore excluded.

**Table 3:** Pearson correlations for study variables

N		DTA	AttachAnxiety	AttachAvoidance
TTO_wheel_	1959	.00	− .03	− .05*
TTO_own_	2023	.03	− .17***	− .13***
VAS_wheel_	928	− .13***	.05	− .08*
VAS_own_	928	− .13** §	− .15***	− .15***

*p < .05 **p < .01 ***p < .001

DTA = Death Thought Accessibility, TTO_wheel_ = TTO score for the state ‘being in a wheelchair’ only for consistent respondents, TTO_own_ = TTO score for ‘your own health today’, only for consistent respondents, VAS_wheel_ = VAS score for the state ‘being in a wheelchair,’ VAS_own_ = VAS score for the state ‘your own health today’. The attachment measures indicate worse attachment: AttachAnxiety = attachment anxiety, AttachAvoidance = attachment avoidance. § data from study 2, N = 466

### Strengths

We were able to show a DTA difference between the MS and control conditions, suggesting that the absence of an elevated DTA in the TTO condition is real. Furthermore, the low rate of inconsistent TTO responses is also a strength as it shows good data quality.

### Relevance

DTA was unrelated to the TTO scores for the hypothetical wheelchair state suggesting that DTA might have limited impact on national health valuation studies. This conclusion is qualified by the online nature of our study without interviewers, without using severe hypothetical health states and no direct comparisons with dead. As attachment is weakly associated with TTO and VAS measures for ‘self-experienced health’, it needs to be considered, 1) when measures of self-experienced health are used in national value sets, or 2) when scores are elicited directly from patients [50]. The VAS’s sensitivity to DTA suggest that the VAS captures a broader psychological concept than the TTO, despite that both methods attempt to quantify health. The latter may inform policy making.

### Future work

In this online context, the DTA differences between MS and control conditions were small. In contrast, the TMT literature showed larger differences in classroom or individual settings. Therefore, our survey needs replication in such a setting. To suppress the rational character of the TTO task, a study comparing health states with dead, e.g., a moderate and severe health state, together with a MS and control group, would be feasible. One might also test the presence or absence of an interviewer or assess DTA in a national valuation study.

## Electronic supplementary material

Below is the link to the electronic supplementary material.

Supplementary file1 (DOCX 17 KB)

## Appendix: Measurement of unconscious and conscious defenses

### Unconscious cultural worldview defense strength: agreement with doctor

#### Rationale

Cultural values are strengthened when unconscious death thoughts occur. For instance, respondents may become more patriotic in the presence of unconscious death thoughts. An analogous route is taken in this paper. Respondents are randomized to a MS and neutral condition. We choose to seek if ‘prolonging life’ is a value used in cultural worldview defense. Respondents read a description of a patient in coma and judge a medical doctor who argues for withholding life support. A less favourable judgement of the doctor is taken to indicate a higher value of the value ‘prolonging life.’

#### Description of a coma patient

“*After an accident, a patient is now in coma and taken care of in the intensive care input. The patient is being kept alive via tubes. A tube for artificial feeding and a tube for urinating. The patient is on an artificial breathing apparatus. The family considers how long this can go on since nothing has changed for 8 days.*

*A medical doctor comes and argues in favour of withholding life support because of poor prospects after continuation of life support. It is unlikely that the patient will survive, and even then, severe brain damage is expected. The doctor proposes to set a date to end life support after which the patient will eventually die.*”

#### Agreement with the doctor

Next, the level of agreement with the medical doctor is measured. Five questions are used to measure the level of agreement, “do you find the doctor likeable”, “do you think he is intelligent”, “do you think he is knowledgeable”, “do you agree with the doctor”, “to what extent do you think the same as the doctor”. Each question is answered on a 9-point scale ranging from 1 (not at all) to 9 (very much). Responses were summed to form a single index ranging from 5 to 45 [17].

#### Results

Results are showed in Table S1. The difference between the MS and control condition is not significant: F(1,697) = 1.6, *p* = 0.2. Since significance was considered a prerequisite for using this measure, further analyses on unconscious defenses were abandoned.

### Conscious defense strength: emotional lability inventory

#### Rationale

The conscious defense measure “gives participants an opportunity to bias their emotional responses to appear to be the type of person who will not die at an early age. The bias occurs after reading a cover story stating that a tremendous amount of change in emotion (half the participants read that people who experience too little change in emotion) die at an early age.”[24, 38] Respondents are then asked to fill out an emotional lability questionnaire.

#### Medical cover story

The story states that a series of investigations in the prominent medical journal ‘The New England Journal of Medicine’ has determined that people who experience a tremendous amount of change in emotion (half the participants read that people who experience too little change in emotion) and therefore score high (low) on the next questionnaire, die at an early age. It has been found that strongly (or weakly) variable emotions lead to a higher secretion of substances that burden the heart. These substances (epinephrine and norepinephrine) also lead to too much cholesterol in the veins and arteries.

Furthermore, there are indications that in people with strongly (or weakly) variable emotions the immune system is suppressed causing strongly (weakly) emotional people to be more sensitive to several life-threatening diseases such as cancer. It is therefore clearly proven that people with a high (low) score on the next questionnaire live significantly shorter than people who score low (high). In this study we would like to know how emotional lability is related to other questionnaires, as used in this study [24, 38].

#### Emotional lability measure

Participants subsequently completed a series of 14 questions about the extent to which they experience general specific emotions, e.g., on an average day, how often do you feel your heart pounding?” and “on an average day, how often do you have strong emotional reactions [52]. Next, specific affective reactions on an average day were assessed with 10 questions e.g., “on an average day, how often do you feel: joyous, angry, frustrated, exhilarated, disgusted, excited, devastated, scared, euphoric, and surprised?” Participants responded to these questions using 11-point scales ranging from 1 (never experiencing the emotion) to 11 (experiencing the emotion virtually all the time).

#### Results

Table S2 shows the results for the conditions where a high or low emotionality indicates a bias towards being the person who does not die at a younger age. The pattern is as expected: a lower lability score where high lability is bad, and a higher lability score when low lability is bad. However, the difference is not significant, F(1,701) = 1.9, *p* = 0.17. Since we considered a significant difference as a prerequisite for using this measure, further analyses on the conscious defenses were abandoned.
