# Supplementary material for: Unconscious death thoughts: Do they play a role in time trade-off and visual analogue scale scores for health?
Source: Qual Life Res. 2026 Feb 3;35(3):60. doi: 10.1007/s11136-025-04145-0 (PMC12868079; doi:10.1007/s11136-025-04145-0)
Supplement: Supplementary file 1 — Supplementary file1 (DOCX 17 KB) [file 11136_2025_4145_MOESM1_ESM.docx]

Table S1: Unconscious defense strength for the value ‘prolonging life’ by condition

| Condition |  | Mean (SD) |  | N |
| --- | --- | --- | --- | --- |
| MS |  | 35.15 (6.99) |  | 345 |
| Control |  | 34.46 (7.28) |  | 354 |

*MS = Mortality Salient, Control = television control*

Table S2: Emotional lability by condition

| Condition |  | Mean (SD) |  | N |
| --- | --- | --- | --- | --- |
| High lability  is bad |  | 7.02 (1.88) |  | 356 |
| Low lability  is bad |  | 7.22 (1.88) |  | 347 |

*MS = Mortality Salient, Control = television control*
